# Excreting and non-excreting grasses exhibit different salt resistance strategies

**DOI:** 10.1093/aobpla/plu038

**Published:** 2014-07-04

**Authors:** Muhammad Moinuddin, Salman Gulzar, Muhammad Zaheer Ahmed, Bilquees Gul, Hans-Werner Koyro, Muhammad Ajmal Khan

**Affiliations:** 1 Institute of Sustainable Halophyte Utilization (ISHU), University of Karachi, Karachi 75270, Pakistan; 2 Institute of Plant Ecology, Justus-Liebig University Gießen, Gießen D-35392, Germany; 3 Shell Professorial Chair of Sustainable Development, Department of International Affairs, College of Arts and Sciences, Qatar University, PO Box 2713, Doha, Qatar

**Keywords:** C–N balance, compatible solutes, halophytic grasses, ion homoeostasis, Na^+^ flux, nitrogen-use efficiency.

## Abstract

The combination of traits that makes a plant successful under saline conditions varies with the type of plant and its interaction with the environmental conditions. Knowledge about the contribution of these traits towards salt resistance in grasses has great potential for improving the salt resistance of conventional crops. We attempted to identify differential adaptive response patterns of salt-excreting versus non-excreting grasses. More specifically, we studied the growth, osmotic, ionic and nutrient (carbon/nitrogen) relations of two salt-excreting (*Aeluropus lagopoides* and *Sporobolus tremulus*) and two non-excreting (*Paspalum paspalodes* and *Paspalidium geminatum*) perennial C_4_ grasses under non-saline and saline (0, 200 and 400 mM NaCl) conditions. Growth and relative growth rate decreased under saline conditions in the order *P. geminatum* > *S. tremulus* = *A. lagopoides* > *P. paspalodes*. The root-to-shoot biomass allocation was unaffected in salt-excreting grasses, increased in *P. paspalodes* but decreased in *P. geminatum*. Salt-excreting grasses had a higher shoot/root Na^+^ ratio than non-excreting grasses. K^+^, Ca^2+^ and Mg^2+^ homoeostasis remained undisturbed among test grasses possibly through improved ion selectivity with rising substrate salinity. Salt-excreting grasses increased leaf succulence, decreased *ψ*_s_ and xylem pressure potential, and accumulated proline and glycinebetaine with increasing salinity. Higher salt resistance of *P. paspalodes* could be attributed to lower Na^+^ uptake, higher nitrogen-use efficiency and higher water-use efficiency among the test species. However, *P. geminatum* was unable to cope with salt-induced physiological drought. More information is required to adequately document the differential strategies of salt resistance in salt-excreting and non-excreting grasses.

## Abstract (precis)

Salt marsh grasses are adapted to thrive under saline conditions by various combinations of traits. Some researchers suggested that salt excreting grasses would differ from non-excreting ones in these traits. However, little is known about the differential responses between these plant types. Here, we compared the growth and physiology of salt excreting and non-excreting grasses. Differences were found between the two grass types in leaf water content, accumulation of organic compounds and Na^+^ distribution which appeared to be linked with salt excretion. Additional studies on a number of halophytic grasses could help to identify key traits for salt resistance.

## Introduction

Halophytic grasses in arid and semi-arid areas dominate salt marsh vegetation where soil salinity varies between 1 and 30 dS m^−1^ and occasionally at or above seawater salinity (Gulzar and Khan 1994). Considerable variations in salinity, moisture and nutrients allow different species to occupy distinct ecological niches. Phylogenetic studies indicate that salt tolerance evolved more than 70 times in grasses and is supported by studies on intraspecific variations in physiological, morphological and biochemical responses under saline conditions (Bennett *et al*. 2013).

Salt resistance is a complex trait (Flowers and Colmer 2008; Flowers *et al*. 2010) and represents a continuum between glycophytes and halophytes (Bonales-Alatorre *et al*. 2013). Decreased water uptake, ion toxicity, nutrient imbalance, reduced photosynthesis and subsequent production of reactive oxygen species usually lead to growth reduction under saline conditions. Reduced biomass allocation to shoot versus root is reported to ensure long-term survival by improved water and ion balance and reduced salt accumulation in the rhizosphere (Passioura *et al*. 1992). Grasses generally tend to maintain low Na^+^ in aboveground tissues in comparison with dicotyledonous halophytes by restricting Na^+^ uptake (Flowers and Colmer 2008). Salt tolerance in grasses was found to be negatively correlated with shoot [Na^+^] (Marcum *et al*. 1998). The higher K^+^/Na^+^ selectivity and potassium-use efficiency of grasses compared with most dicotyledonous species (Flowers and Colmer 2008) is essential for survival under saline conditions. More than 30 grass species from about 16 genera mostly in the tribes Chlorideae, Sporoboleae and Aeluropodeae are known to remove salt from the leaf surface by means of bi-cellular epidermal salt glands (Liphschitz and Waisel 1974; Marcum 2001; Kobayashi and Masaoka 2008; Lefèvre *et al*. 2009). Salt resistance has been associated often with efficient salt excretion from leaves (Marcum *et al*. 1998) and also with increased water-use efficiency (WUE), a typical feature of C_4_ grasses (Naidoo *et al*. 2012).

Plants need to maintain water uptake through osmotic adjustment under saline conditions and are known to accumulate carbon (C)-rich (sugars, sugar alcohols) and nitrogen (N)-rich (proline, glycinebetaine, trigonelline) organic osmolytes to counterbalance salt entry into the plant (Munns 2002, 2011; Koyro *et al*. 2011). Accumulation of N-rich compounds also makes aboveground tissues more palatable for herbivores (Elser *et al*. 2000). However, increased salinity may hinder N uptake because of competition with Cl^−^ and Na^+^ accumulation (Taiz and Zeiger 2006) and reduce C uptake because of reduction of CO_2_/H_2_O gas exchange rates. Therefore, salinity resistance of halophytic species was also associated with efficient N allocation towards synthesis of organic compounds (Geissler *et al*. 2009) which in turn is linked with processes for C assimilation into biomass (Hussin *et al*. 2013).

Salt tolerance is a complex phenomenon which is a manifestation of multigenic traits at the whole-plant level. However, little information is available on small-scale differences among salt-excreting (*Aeluropus lagopoides* and *Sporobolus tremulus*) and non-excreting (*Paspalum paspalodes* and *Paspalidium geminatum*) C_4_ salt marsh grasses. The salt-excreting species are characteristically found in more saline sandy soils (ECe > 1.5 dS m^−1^) while the non-excreting ones appear to prefer clayey, less salty substrates (ECe < 1.0 dS m^−1^). To the authors knowledge no salt tolerance studies have been carried out on latter three grass species and previous work on salt tolerance of *A. lagopoides* (Gulzar *et al*. 2003) was related only to growth and water status but not to its nutrient status or synthesis of compatible osmolytes under saline stress. The present research is an attempt to scale up from the physiological/biochemical level to understand the whole-plant salt resistance mechanisms of four C_4_ grasses growing naturally in saline marshes. We hypothesize that salt-excreting grasses will differ from non-excreting ones in terms of (i) growth, (ii) water relations, (iii) ion regulation and (iv) nitrogen-use efficiency (NUE). The results should also provide some explanations about their distribution in natural populations. Therefore, we compared the relative salt resistance in terms of growth, water and ion relations, C/N ratios and NUE of C_4_ salt excreting (*A. lagopoides* and *S. tremulus*) (Chloridoideae) and non-excreting (*P. paspalodes* and *P. geminatum*) (Panicoideae) grasses.

## Methods

### Experimental conditions

Ramets of *P. paspalodes* and *P. geminatum* were collected from Korangi, Karachi (24°51′03.2″N; 67°05′60.4″E), while *A. lagopoides* and *S. tremulus* were collected from Manora Creek near Sandspit, Karachi (24°49′06.70″N; 66°56′06.80″E). Tillers were potted in plastic pots (26 cm high×20 cm diameter) in sand culture and watered daily to a constant volume by sub-irrigation with half strength Hoagland solution (Epstein 1972) to establish for 1 month. Salt treatments (0, 200 and 400 mM NaCl) were introduced gradually with 100 mM NaCl increments every 24 h to avoid osmotic shock. Final concentrations in trays used for sub-irrigation were maintained daily by adding distilled water to compensate for evaporation. In plastic pots this was achieved by flushing soil from above with the respective nutrient solution at 3–4-day intervals allowing them to drip from below. The treatment solutions were replaced every fifth day. Plants were cut at 15 cm above the soil surface when final salt concentrations were reached which were maintained for another 45 days before the final harvest.

### Growth parameters

Plants were carefully removed from the soil and roots were washed with the respective solution and then dipped twice in distilled water for a few seconds and wiped with tissue paper. Roots were separated from shoots and then measured for fresh weight. Plant samples were placed in a microwave oven for about 6 min to determine dry weight (Popp *et al*. 1996). A half-filled 100-mL beaker with distilled water was also placed alongside the samples to avoid burning. Plant samples were allowed to cool down in a desiccator before measuring the dry weights. Relative growth rate (RGR) was calculated using the formula:

$$\text{RGR}\;(\text{g}\;{\text{g}}^{-1}\;{\text{day}}^{-1})=\frac{\left(\text{ln}W_{2}-\text{ln}W_{1}\right)}{\left(t_{2}-t_{1}\right)}$$

where *W*_1_ and *W*_2_ are the initial and final dry weights whereas *t*_1_ and *t*_2_ are the initial and final time in days from the start of salinity treatments.

### Water relations

Leaf succulence on a dry weight basis was measured using the equation:

$$\text{Succulence}\;(\text{g}\;{\text{H}}_{2}\text{O}\;{\text{g}}^{-1}\;\text{DW})=\frac{\left(\text{FW}-\text{DW}\right)}{\text{DW}}$$

Relative water content (RWC) was found out with the help of the formula:

$$\text{RWC}\;(\%)=\frac{\left(\text{FW}-\text{DW}\right)}{\left(\text{TW}-\text{DW}\right)}\times \;100$$

where FW is the fresh weight; DW the dry weight and TW the turgid weight of leaves after rehydration in distilled water for 24 h at room temperature (∼25 °C).

Leaf osmotic potential was calculated using van't Hoff equation (Kramer and Boyer 1995) on osmolality of expressed leaf sap measured by a vapour pressure osmometer (VAPRO-5520; Wescor Inc., Logan, UT, USA) (Gucci *et al*. 1991). Xylem pressure potential (XPP) was measured on excised stems with a plant water status console Model 1000 (PMS Instrument Co., Albany, NY, USA). Instantaneous WUE was calculated from the rate of CO_2_ fixation (*A*) which was measured using a Li-6400XT portable photosynthesis system (LICOR Biosciences) per amount of water transpired (*E*) from the leaf surface as

$$\text{WUE}\;(\mu \text{mol}\;\;{\text{CO}}_{2}\;{\text{mmol}}^{-1}\;\;{\text{H}}_{2}\text{O})=\frac{A}{E}$$

### Cation contents (Na^+^, K^+^ Ca^2+^ and Mg^2+^)

Hot-water extracts were prepared with homogenized finely ground dry plant material in deionized water at 100 °C (Khan *et al*. 2000) in capped Pyrex test tubes. Soluble Na^+^, K^+^, Ca^2+^ and Mg^2+^ in shoot and root were determined on dilutions of the hot water extracts by atomic absorption spectrometry (AA-700; Perkin Elmer, Santa Clara, CA, USA).

### Selectivity of K^+^, Ca^2+^ and Mg^2+^ over Na^+^

Selective absorption (SA) and selective transport (ST) of K^+^, Ca^2+^ and Mg^2+^ over Na^+^ were calculated according to Wang *et al*. (2005) as follows:

$${\text{SA}}_{X}=\frac{(X/{\text{Na}}^{+}{)}_{\mathrm{root}}}{(X/{\text{Na}}^{+}{)}_{\mathrm{medium}}};\;{\text{ST}}_{X}=\frac{(X/{\text{Na}}^{+}{)}_{\mathrm{shoot}}}{(X/{\text{Na}}^{+}{)}_{\mathrm{root}}}$$

where ‘*X*’ stands for K^+^, Ca^2+^ or Mg^2+^

### Organic osmolytes

Total soluble sugars were determined using the method of Yemm and Willis (1954). A 1-ml dilution of hot water extract in distilled water was added to 5 mL Anthrone's reagent in a Pyrex test tube and incubated in a water bath at 100 °C for 30 min. The reaction was terminated in an ice bath and the absorbance was recorded at 630 nm with a spectrophotometer (Beckman DU-530 spectrophotometer; Beckman Coulter Inc., USA). Proline was determined according to Bates *et al*. (1973) on 200 µL of hot water extract made up to 2 mL with distilled water which was mixed with ninhydrin : glacial acetic acid (1 : 1 v/v) and incubated at 100 °C for 1 h. The reaction was terminated on an ice bath and the chromophore was extracted with 4 mL toluene. Proline content was measured at 520 nm on a spectrophotometer (Beckman DU-530 spectrophotometer, Beckman Coulter Inc.). Glycinebetaine was determined by using dilutions of hot water extract in 50 mM potassium dihydrogen phosphate buffer (4.5 pH), the mobile phase for the high-performace liquid chromatography. The samples were filtered through a 0.45-µm membrane filter before injecting into a Nucleosil HPLC Column (4.6 × 250 mm) on a JASCO Intelligent UV/VIS HPLC system (JASCO, Japan) at a flow rate of 1.2 mL min^−1^ at 25 °C. Estimations were based on standard curves of 1, 10 and 100 mmol dilutions of glycinebetaine (Khan *et al*. 1999).

### Carbon, nitrogen analyses and NUE

Carbon and nitrogen were determined on 0.4 mg fine ground dry shoot material mixed with tungsten trioxide (catalyst) wrapped in a tin foil boat. The sample boats were loaded on the auto sampler of a CNS elemental analyzer Vario EL III equipped with a thermal conductivity detector (Elementar Analysensysteme GmbH, Germany). Helium (99.99 % purity) was the carrier gas and oxygen (99.999 % purity) was used for oxidation of plant material when required. Nitrogen-use efficiency was calculated following the method of Hussin *et al*. (2013):

$$\text{NUE (gDW}\;{\text{gN}}^{-1})=\frac{\text{whole}\;\text{plant}\;\text{DW}}{\text{total}\;\text{shoot}\;\text{N}}$$

### Statistical analyses

Statistical analysis was carried out using SPSS Ver. 11.0 for Windows (SPSS Inc., Chicago, IL, USA) (SPSS 2006). Two-way analysis of variance (ANOVA) was used to test for significant differences among species, salinity and their interactions. One-way ANOVA was used to reveal significant differences across salinity treatments within individual species while a post hoc Bonferroni test was used to test for significant differences between individual treatment means.

## Results

### Growth parameters

**Figure 1.**
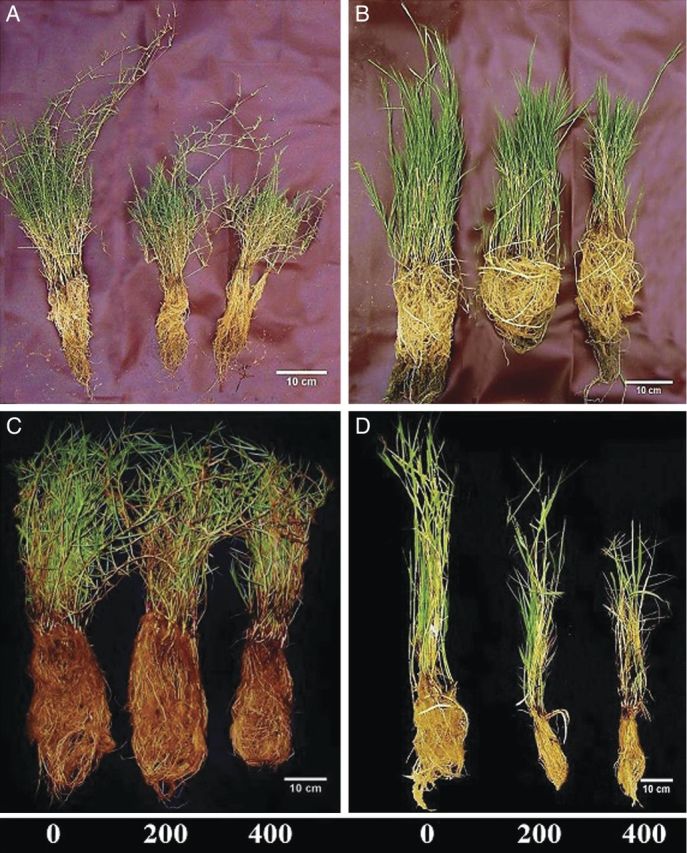
Relative growth of (A) *A. lagopoides*, (B) *S. tremulus*, (C) *P. paspalodes* and (D) *P. geminatum* grown under increasing salinity treatments (0, 200 and 400 mM NaCl).

**Figure 2.**
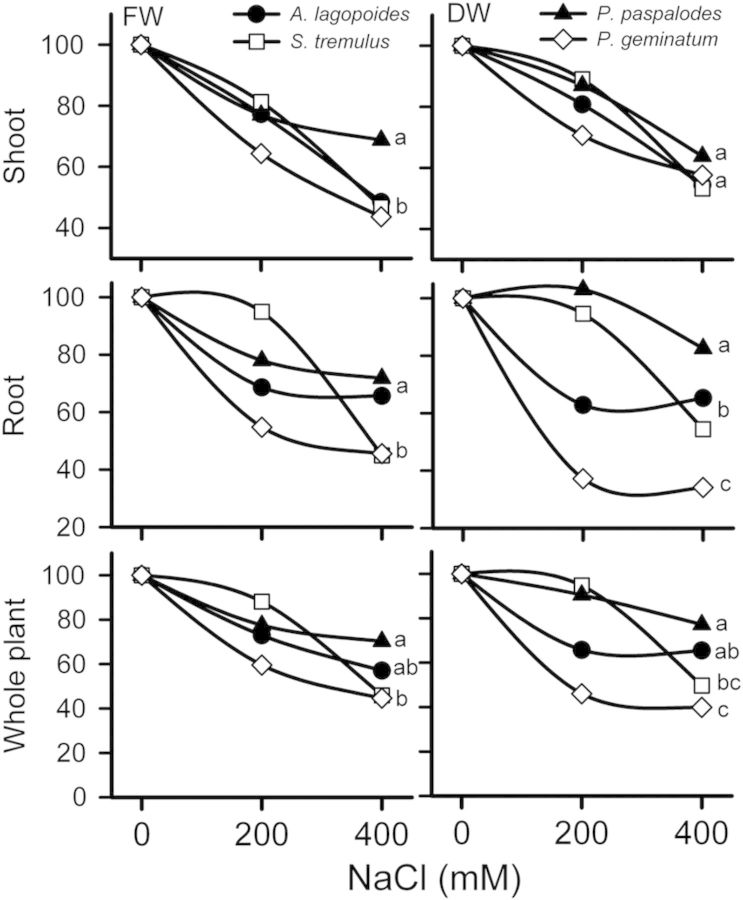
Percent change in the fresh weight (FW) and dry weight (DW) at shoot, root and whole-plant levels of the salt-excreting (*A. lagopoides*, *S. tremulus*) and non-excreting (*P. paspalodes*, *P. geminatum*) grasses grown under increasing salinity treatments (0, 200 and 400 mM NaCl). Different letters represent significant differences among species at *P* < 0.05 (Bonferroni test).

**Figure 3.**
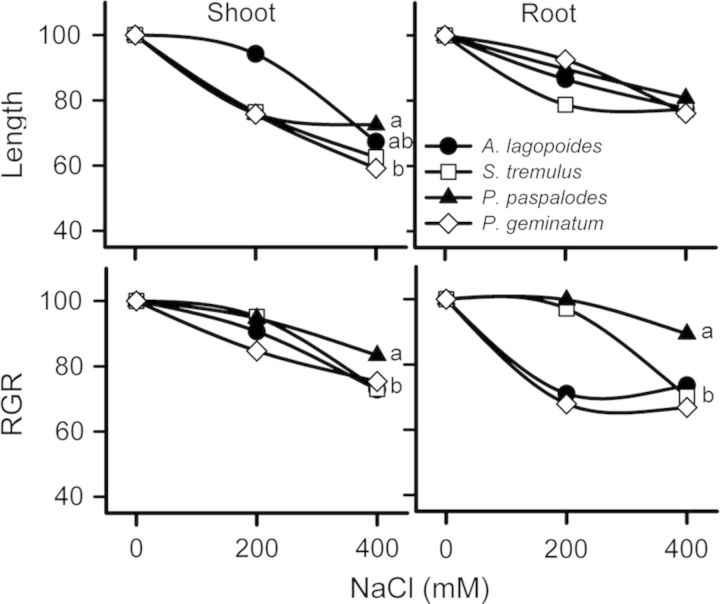
Percent change in the length and RGR of aboveground and belowground parts of the salt-excreting (*A. lagopoides*, *S. tremulus*) and non-excreting (*P. paspalodes*, *P. geminatum*) grasses grown under increasing salinity treatments (0, 200 and 400 mM NaCl). Different letters represent significant differences among species at *P* < 0.05 (Bonferroni test).

Two-way ANOVA indicated a significant decrease in shoot and root biomass, shoot and root length and RGRs in all grass species (*P* < 0.001) with increase in salinity (*P* < 0.05) (Figs. 1–3). Shoot length was affected more than root growth in all grasses (Fig. 3). Dry mass of *P. geminatum* was reduced by 60 % in 400 mM NaCl compared with those grown in 0 mM NaCl. Plant biomass reduction was 35 and 50 % for the salt-excreting grasses (*A. lagopoides* and *S. tremulus*), respectively, but only 23 % for *P. paspalodes* (Fig. 2). The root-to-shoot biomass allocation ratio was quite variable among species (*F* = 9.2; *P* < 0.001; Table 1) which registered a salt-stimulated increase in *P. paspalodes* (*P* < 0.05). Salt-excreting grasses displayed lower and generally invariable root-to-shoot biomass allocation ratios than non-excreting ones, whereas a marked reduction (50 %) in the root-to-shoot biomass ratio was noted under saline conditions in *P. geminatum* (*P* < 0.05; Table 1). Relative growth rates differed significantly among species in root (*F* = 48.19; *P* < 0.0001) and shoot (*F* = 20.39; *P* < 0.0001). Similarly, RGR also varied significantly with increasing salinity in root (*F* = 13.73; *P* < 0.0001) and shoot (*F* = 13.73; *P* < 0.0001) of all test species. Root RGR remained unchanged (*F* = 15.50; *P* > 0.05) only in *P. paspalodes* with increase in salinity (Fig. 3).

**Table 1.** Root-to-shoot dry biomass allocation of salt-excreting (*A. lagopoides*, *S. tremulus*) and non-excreting (*P. paspalodes*, *P. geminatum*) grasses grown under increasing salinities (0, 200 and 400 mM NaCl). Numbers are means (±SE) at each salinity level (*n* = 3). Different letters represent significant differences among species at *P* < 0.05 (Bonferroni test).

NaCl (mM)	Salt excreting		Non-excreting	
	*A. lagopoides*	*S. tremulus*	*P. paspalodes*	*P. geminatum*
0	0.36 ± 0.02a	0.56 ± 0.07a	0.58 ± 0.05a	1.10 ± 0.09a
200	0.28 ± 0.01b	0.59 ± 0.04a	0.71 ± 0.16b	0.56 ± 0.09b
400	0.44 ± 0.02c	0.57 ± 0.02a	0.74 ± 0.24b	0.66 ± 0.08b

### Water relations

**Figure 4.**
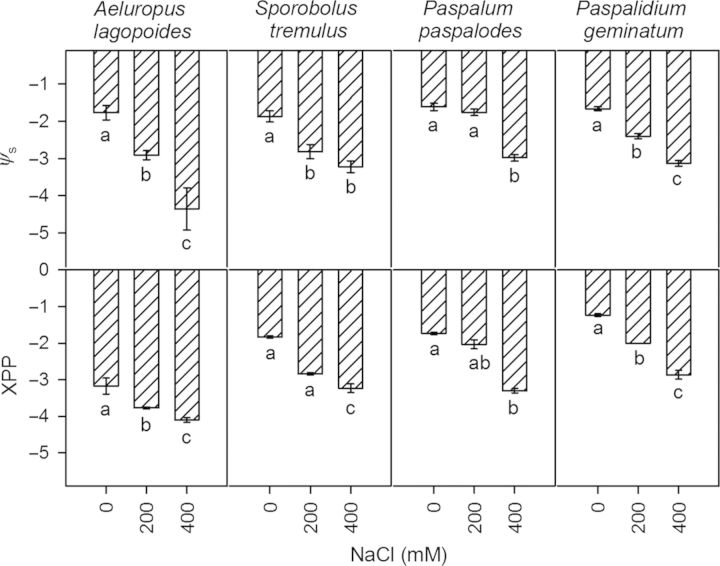
Osmotic potential (*ψ*_s_, MPa) and XPP (MPa) of the salt-excreting (*A. lagopoides*, *S. tremulus*) and non-excreting (*P. paspalodes*, *P. geminatum*) grasses grown under increasing salinity treatments (0, 200 and 400 mM NaCl). Bars are means (±SE) at each salinity level (*n* = 3). Different letters represent significant differences among species at *P* < 0.05 (Bonferroni test).

Leaf succulence increased in salt-excreting grasses and it decreased in non-excreting grasses with increase in NaCl concentrations (Table 2). Relative water content increased in salt-excreting grasses, remained unchanged in *P. paspalodes* but decreased sharply in *P. geminatum*. Water-use efficiency of salt-excreting grasses was unaffected while it increased (*P* < 0.0001) in *P. paspalodes* and declined (*P* < 0.0001) in *P. geminatum* (Table 2) with increase in NaCl concentrations. Leaf osmotic potential (*ψ*_s_) and XPP showed significant differences among species (*P* < 0.0001) and were particularly lower in the salt-excreting grasses (Fig. 4). Effects of salinity and species × salinity interactions were highly significant for *ψ*_s_ (*P* < 0.001, *P* < 0.05 respectively) and XPP (*P* < 0.001).

**Table 2.** Leaf succulence (g g^−1^ dry weight), relative water content (RWC; %) and WUE (µmol CO_2_ mmol^−1^ H_2_O) of salt-excreting (*A. lagopoides*, *S. tremulus*) and non-excreting (*P. paspalodes*, *P. geminatum*) grasses grown under increasing salinities (0, 200 and 400 mM NaCl). Numbers are means (±SE) at each salinity level (*n* = 3). Different letters represent significant differences among species at *P* < 0.05 (Bonferroni test).

NaCl (mM)	Salt excreting		Non-excreting	
	*A. lagopoides*	*S. tremulus*	*P. paspalodes*	*P. geminatum*
Leaf succulence (g g^−1^ dry weight)				
0	1.41 ± 0.04a	1.47 ± 0.08a	2.57 ± 0.09a	2.53 ± 0.20a
200	1.73 ± 0.05b	1.90 ± 0.03b	2.45 ± 0.02a	1.98 ± 0.10b
400	1.83 ± 0.07b	1.72 ± 0.15b	2.10 ± 0.02b	1.62 ± 0.16b
Leaf RWC (%)				
0	58.4 ± 0.37a	75.7 ± 4.29a	69.7 ± 3.12a	65.4 ± 0.79a
200	74.8 ± 3.55b	84.8 ± 1.55b	70.0 ± 0.35a	61.9 ± 2.13a
400	81.9 ± 3.62c	73.0 ± 3.65a	63.1 ± 0.28a	50.0 ± 2.53b
WUE (µmol CO_2_ mmol^−1^ H_2_O)				
0	3.71 ± 0.06a	3.89 ± 0.17a	3.04 ± 0.51a	4.38 ± 0.28a
200	3.59 ± 0.12a	4.36 ± 0.16a	3.85 ± 1.02a	0.54 ± 0.18b
400	3.62 ± 0.61a	3.23 ± 0.09a	5.62 ± 0.42b	0.25 ± 0.18b

### Cation contents (Na^+^, K^+^ Ca^2+^ and Mg^2+^)

**Figure 5.**
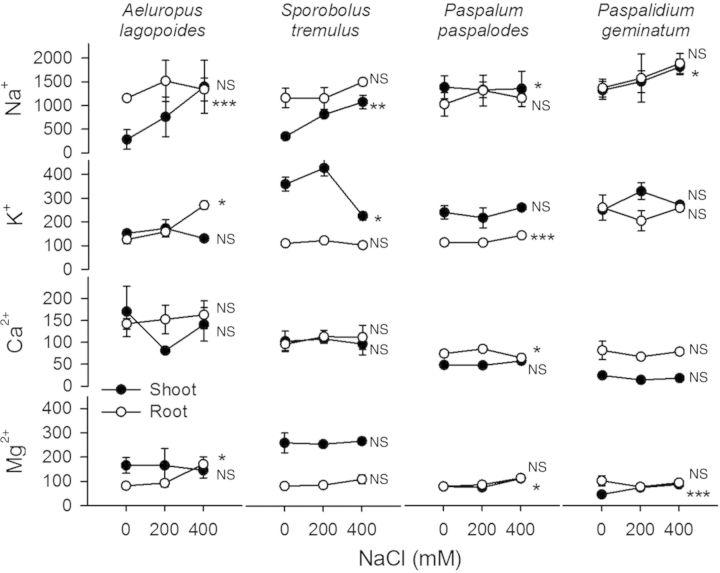
Cation (Na^+^, K^+^, Ca^2+^ and Mg^2+^) content (mmol kg^−1^ dry weight) in shoot and root of the salt-excreting (*A. lagopoides*, *S. tremulus*) and non-excreting (*P. paspalodes*, *P. geminatum*) grasses grown under increasing salinity treatments (0, 200 and 400 mM NaCl). Symbols indicate means (±SE) at each salinity level (*n* = 3). Asterisks (*, ** and ***) represent significant differences between cation contents at *P* < 0.05, 0.01 and 0.001 respectively; NS indicates non-significant differences among salinity treatments by one-way ANOVA.

**Figure 6.**
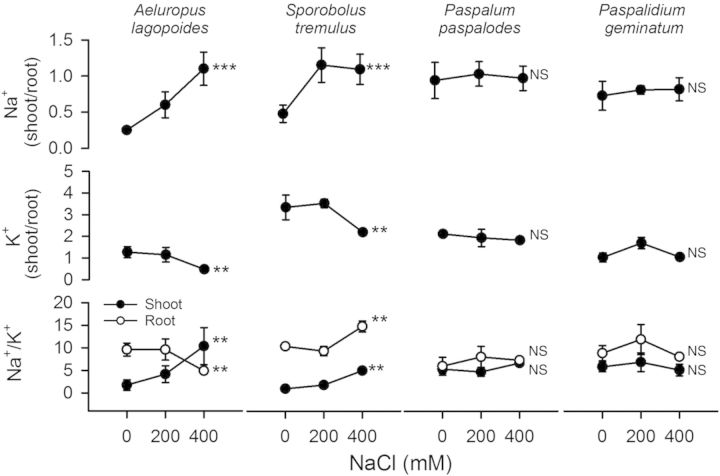
Shoot/root ratios of Na^+^ and K^+^ and Na^+^/K^+^ ratios of the salt-excreting (*A. lagopoides*, *S. tremulus*) and non-excreting (*P. paspalodes*, *P. geminatum*) grasses grown under increasing salinity treatments (0, 200 and 400 mM NaCl). Symbols indicate means (±SE) at each salinity level (*n* = 3). Asterisks (** and ***) represent significant differences among cation contents at *P* < 0.01 and 0.001 respectively; NS indicates non-significant differences among salinity treatment by one-way ANOVA.

Na^+^ content of shoot in all test grasses increased substantially (*F* = 16.89; *P* < 0.001) compared with root with increasing salinity. K^+^ was generally higher in shoots than in roots but declined sharply in *S. tremulus* (*F* = 7.10; *P* < 0.01) shoots at 400 mM NaCl (Fig. 5). Shoot/root Na^+^ ratios also increased with concomitant decreases in shoot/root K^+^ ratios in salt-excreting grasses but remained unchanged in non-excreting grasses. Shoot Na^+^/K^+^ ratios increased significantly (*P* < 0.05) in salt-excreting grasses at 400 mM NaCl but did not vary in the non-excreting grasses (Fig. 6). K^+^, Ca^2+^ and Mg^2+^ content in shoot and root of our test grasses under saline treatments was not lower than their respective non-saline controls.

### Selectivity of K^+^, Ca^2+^ and Mg^2+^ over Na^+^

Selective absorption of K^+^, Ca^2+^ and Mg^2+^ against Na^+^ increased in all test species (*P* < 0.001) and with increasing NaCl concentrations (*P* < 0.001; Table 3). Selective transport of K^+^ and Mg^2+^ against Na^+^ towards shoot varied among test species (*P* < 0.001) and salinity treatments (*P* < 0.05) except for ST of Ca^2+^which was unaffected by salinity (Table 3).

**Table 3.** SA and ST of K^+^/Na^+^, Ca^2+^/Na^+^ and Mg^2+^/Na^+^ in shoot and root of salt-excreting (*A. lagopoides*, *S. tremulus*) and non-excreting (*P. paspalodes*, *P. geminatum*) grasses grown under increasing salinities (0, 200 and 400 mM NaCl). Numbers are means (±SE) at each salinity level (*n* = 3). Different letters represent significant differences among species at *P* < 0.05 (Bonferroni test).

NaCl (mM)	K^+^/Na^+^		Ca^2+^/Na^+^		Mg^2+^/Na^+^	
	SA	ST	SA	ST	SA	ST
*A. lagopoides*						
0	2.0 ± 0.5a	4.9 ± 1.1a	5.4 ± 0.4a	2.6 ± 0.4a	7.5 ± 0.5a	5.0 ± 0.6a
200	3.6 ± 0.9a	2.2 ± 0.9b	40.8 ± 2.6b	1.2 ± 0.2a	63.4 ± 14.6b	3.3 ± 0.6b
400	7.3 ± 1.5b	0.5 ± 0.1c	100.3 ± 30.9c	1.9 ± 0.7a	254.1 ± 70.5c	2.0 ± 0.8b
*S. tremulus*						
0	3.0 ± 1.1a	10.8 ± 1.9a	4.0 ± 1.3a	2.2 ± 0.6a	8.0 ± 1.8a	5.3 ± 0.8a
200	7.0 ± 1.3b	4.9 ± 1.1b	42.2 ± 10.8b	0.7 ± 0.1a	76.8 ± 17.1b	2.8 ± 0.5b
400	6.4 ± 0.9b	3.1 ± 0.9b	56.2 ± 15.7b	2.7 ± 1.2a	132.5 ± 25.9c	3.4 ± 0.8b
*P. paspalodes*						
0	0.9 ± 0.2a	1.6 ± 0.2a	1.0 ± 0.2a	0.5 ± 0.1a	4.4 ± 1.1a	0.8 ± 0.2a
200	3.1 ± 0.4b	1.9 ± 1.0a	8.0 ± 1.6b	0.5 ± 0.0a	32.2 ± 5.7b	0.8 ± 0.0a
400	8.0 ± 1.6c	1.6 ± 0.9a	12.0 ± 1.9b	1.0 ± 0.3a	84.8 ± 11.3c	1.0 ± 0.3a
*P. geminatum*						
0	0.5 ± 0.1a	0.7 ± 0.6a	0.8 ± 0.2a	0.4 ± 0.2a	3.9 ± 0.9a	0.6 ± 0.2a
200	5.0 ± 1.0b	1.6 ± 0.8a	6.0 ± 2.0b	0.2 ± 0.1a	26.4 ± 6.6b	1.0 ± 0.1a
400	10.6 ± 0.6c	0.9 ± 0.1a	9.1 ± 1.3b	0.2 ± 0.0a	43.3 ± 3.9c	1.0 ± 0.1a

### Organic osmolytes

**Figure 7.**
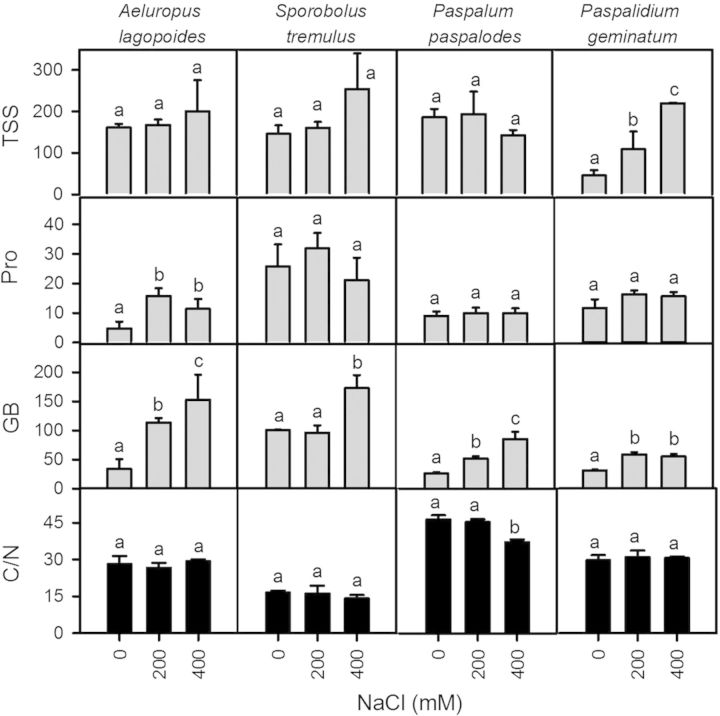
Total soluble sugars (TSS), proline (Pro) and glycinebetaine (GB) in mmol kg^−1^ dry weight, C/N ratio in shoots of the salt-excreting (*A. lagopoides*, *S. tremulus*) and non-excreting (*P. paspalodes*, *P. geminatum*) grasses grown under increasing salinity treatments (0, 200 and 400 mM NaCl). Bars are means (±SE) at each salinity level (*n* = 3). Different letters represent significant differences among species at *P* < 0.05 (Bonferroni test).

Two-way ANOVA indicated no effect of species (*F* = 1.43; *P* = 0.26) and salinity (*F* = 3.09; *P* = 0.06) on total soluble sugars (TSS) (Fig. 7). Total soluble sugars, on a dry weight basis, significantly increased with increasing salinity only in *P. geminatum* (*F* = 6.15; *P* < 0.01). Proline varied significantly among species (*F* = 11.13; *P* < 0.0001) and was higher in salt-excreting grasses (20–30 mmol kg^−1^ dry weight) but was not affected by NaCl (*F* = 2.19; *P* > 0.05). Proline increased significantly only in *A. lagopoides* (*F* = 3.6; *P* < 0.05; Fig. 7). Glycinebetaine varied significantly among species (*F* = 15.07; *P* < 0.0001), with salinity increments (*F* = 18.24; *P* < 0.0001) with generally higher mean values (>150 mmol kg^−1^ dry weight) in the salt-excreting grasses compared with the non-excreting ones.

### Carbon, nitrogen analyses and NUE

**Figure 8.**
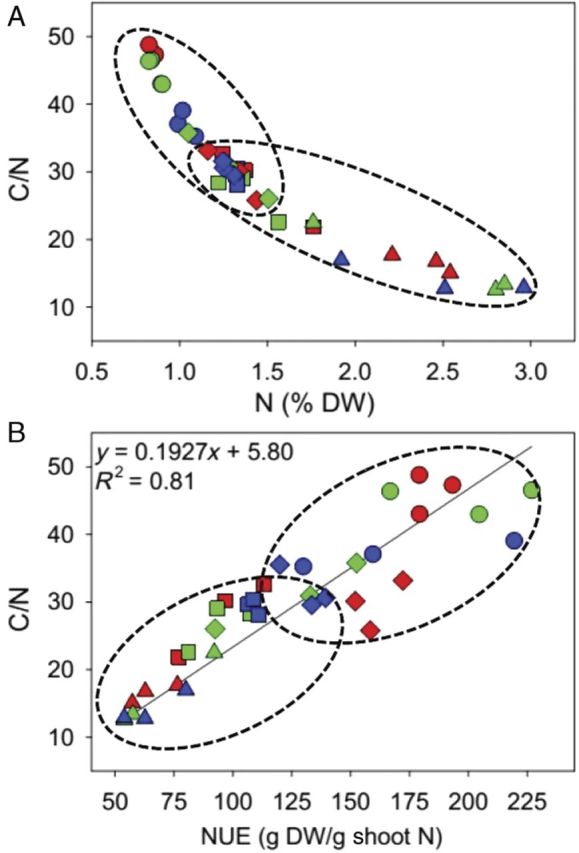
Relationship between C/N ratios and (A) nitrogen (% dry weight) and (B) NUE (g dry weight g^−1^ shoot N) of the salt-excreting (*A. lagopoides* = squares; *S. tremulus* = triangles) and non-excreting (*P. paspalodes* = circles; *P. geminatum* = diamonds) grasses grown under increasing NaCl treatments (0 mM = red; 200 mM = green; 400 mM = blue).

Median nutrient (N and C) concentrations were measured at 38.5 % for C and 1.4 for N with considerable variations in *S. tremulus* (lower C, higher N) and *P. paspalodes* (lower C and N) **[see Supporting Information]**. In general, non-excreting grasses had a high (>30) C/N ratio compared with salt-excreting grasses (Fig. 7) which decreased due to increasing shoot N from 0.8 % in *P. paspalodes* to about the median value of 1.4 % in *P. geminatum* (Fig. 8A). *Sporobolus tremulus* had the highest N at 3 % of dry weight (Fig. 8A). Nitrogen-use efficiency (on dry weight basis) was substantially (*P* < 0.001) higher in non-excreting grasses than in the salt-excreting ones but did not vary with salinity treatments except for a decline in *P. geminatum*. Shoot C/N ratios were linearly correlated (*R*^2^ = 0.81) with NUE across all species and salinity treatments. Salt-excreting grasses displayed lower values for C/N and NUE relative to non-excreting grasses (Fig. 8B; Table 4).

**Table 4.** Summary of key results comparing responses of the salt-excreting (*A. lagopoides*, *S. tremulus*) and non-excreting (*P. paspalodes*, *P. geminatum*) grasses grown under increasing salinity treatments. The direction and number of arrow indicate significant (*P* < 0.05) variations (↑ increase, ↓ decrease, — no change) and degree of variation, respectively.

Parameters	Salt excreting		Non-excreting	
	*A. lagopoides*	*S. tremulus*	*P. paspalodes*	*P. geminatum*
Plant biomass	↓↓	↓↓↓	↓	↓↓↓↓
Root/shoot biomass	—	—	↑	↓
*ψ*_s_ and XPP	↓↓	↓↓	↓	↓
Leaf succulence	↑	↑	↓	↓↓
Relative water content	↑	↑	—	↓
WUE	—	—	↑	↓
Na^+^ shoot/root	↑	↑	—	—
K^+^ shoot/root	↓	↓	—	—
SA—K^+^, Ca^2+^, Mg^2+^/Na^+^	↑	↑	↑	↑
ST—K^+^/Na^+^, Mg^2+^/Na^+^	↓	↓	—	—
Total soluble sugars	—	—	—	↑
Proline	↑	—	—	—
Glycinebetaine	↑↑	↑↑	↑	↑
Carbon/nitrogen	—	—	↓	—
NUE	—	—	—	↓

## Discussion

### Growth and biomass allocation

We found species-specific variations in eco-physiological traits for salt resistance in four perennial C_4_ grasses. However, traits related to ion transport and nutrient allocation on dry weight basis appeared to be related to their excreting versus non-excreting nature. Salinity inhibited the growth of test species in the following order: *P. geminatum*> *S. tremulus*= *A. lagopoides*> *P. paspalodes*. The upper limit of salt resistance in salt-excreting grasses varied from 300 mM NaCl (*Halopyrum mucronatum*; Khan *et al*. 1999) to 1000 mM NaCl (*A. lagopoides* and *Urochondra setulosa*; Gulzar and Khan 2006; Ahmed *et al*. 2013) and in non-excreting grasses it was up to 500 mM NaCl (*Panicum turgidum*, Koyro *et al*. 2013; *Phragmitis karka*, Abideen *et al*. 2014). The growth of *P. geminatum* was inhibited and leaf senesced earlier by salinity compared with other grasses. Reduced biomass production without apparent signs of injury or senescence of salt-excreting grasses in our experiment was recorded in up to 400 mM NaCl. This indicated a plastic response for long-term survival by diverting available energy to ensure survival rather than higher growth and reproduction. *Paspalum paspalodes* proved to be the most salt-resistant grass among the four test species.

Shift in biomass allocation towards root in halophytic grasses could improve their water and nutrient uptake under saline conditions (Donovan and Gallagher 1985; Marcum 1999) and therefore improve their salt resistance (Lee *et al*. 2004). Variation in biomass allocation among species could also be related to their intrinsic growth rate (Hermans *et al*. 2006). The sharp reduction in root-to-shoot biomass ratios in *P. geminatum* with rise in salinity could result in poor resource acquisition under saline conditions to support aboveground tissues and decreased salt exclusion from root surface. The salt-excreting grasses could tolerate saline conditions whereas salinity stimulated root growth of *P. paspalodes*.

### Water relations

Higher salt resistance would require mechanisms to reduce osmotic and toxic ion effects related to salt accumulation (Neumann 1997, 2011). Decreasing *ψ*_s_ and XPP with increasing salinity and a parallel decrease in leaf succulence and WUE (poor stomatal regulation) resulted in a sharp decline in growth of *P. geminatum*. Growth inhibition in salt-stressed plants was attributed to decreased turgor (short term) and wall extensibility (long term) components of water relations (Neumann *et al*. 1988). Differences in WUE of C_4_ grasses under saline conditions also appear to reflect their differential salt resistance (Carmo-Silva *et al*. 2009; Bennett *et al*. 2013). Salt-excreting grasses increased leaf succulence by decreasing *ψ*_s_ and XPP more readily than non-excreting grasses to ensure water uptake under increasing substrate salinity (Touchette *et al*. 2009) and by maintaining WUEs similar to non-saline controls. *Paspalum paspalodes* managed leaf succulence and RWC by limiting salt uptake in shoots at moderate salinity (200 mM NaCl) but higher (400 mM NaCl) salinity reduced tissue water and osmotic potentials. In addition, increase in WUE of *P. paspalodes* appears to buffer adverse effects of salinity increments on its water relations.

### Na^+^ toxicity, ion homoeostasis and selective uptake

Sodium concentrations increased in both root and shoot tissues of test grasses upon exposure to saline conditions except for *P. paspalodes*. Higher shoot Na^+^ partitioning under saline conditions in salt-excreting grasses compared with non-excreting ones in our study appears partly due to their capacity to excrete salts. Naidoo *et al*. (2012) reported that an increase in biomass of *Spartina maritima* in 20 % seawater was mediated through an efficient salt-excreting mechanism in addition to improved photosynthetic efficiency and resource allocation. Salt-excreting grasses grown under warm ambient conditions in this experiment appeared to constitutively downregulate shoot growth possibly to minimize Na^+^ accumulation in the rhizosphere and reduce shoot Na^+^ uptake. Salt resistance of *P. paspalodes* could be attributed to its ability to minimize shoot Na^+^ uptake and by maintaining essential minerals in metabolically active plant tissues (Gorham *et al*. 1986; Marcum and Murdoch 1992; Peng *et al*. 2004; Ahmed *et al*. 2013; Teakle *et al*. 2013).

In most plants, high Na^+^ influx tends to reduce K^+^ absorption and transport which otherwise has numerous roles in plant tissues such as osmotic adjustment, protein synthesis and enzyme activation (Evans and Wildes 1971; Flowers and Läuchli 1983). However, salt-resistant grasses such as *Sporobolus virginicus* (Marcum and Murdoch 1992), *A. lagopoides*, *Sporobolus ioclados*, *U. setulosa* (Gulzar and Khan 2006) and *Zoysia japonica* (Marcum *et al*. 1998) are known to maintain adequate shoot K^+^ levels under saline conditions. Our test grasses did not appear to be K^+^ deficient in spite of a more than five-fold increase in shoot Na^+^ and maintained more than 125 mmol kg^−1^ DW of shoot K^+^ up to 400 mM NaCl.

Potassium homoeostasis appeared to be achieved by high SA of K^+^ over Na^+^ (Bell and O'Leary 2003; Gulzar *et al*. 2003; Wang *et al*. 2009) in our test grasses and has also been related to stelar K^+^ outward rectifiers (SKORs) and KUP-HAK protein channels (Santa-María *et al*. 1997; Gaymard *et al*. 1998). In addition, reduced ST of K^+^ over Na^+^ in salt-excreting grasses under saline conditions could help in maintaining membrane potential for transmembrane movement of essential macronutrients (Bonales-Alatorre *et al*. 2013).

The salt-excreting grasses *A. lagopoides* and *S. tremulus* displayed higher SA and ST of Ca^2+^ over Na^+^ which could result in the higher tissue Ca^2+^ required for efficient salt excretion. Calcium maintains membrane and cell wall integrity (Marschner 1995), is a secondary messenger in many signal transduction pathways and supports growth under saline conditions via improved K^+^ over Na^+^ selectivity (Läuchli and Grattan 2007). Excessive Na^+^ concentration interferes with Ca^2+^ uptake (Grieve and Fujiyama 1987; Dobermann and Fairhurst 2000) which could be offset by maintaining higher Ca^2+^ selectivity at the root level, possibly through H_2_O_2_-activated Ca-permeable channels (Sun *et al*. 2010). Magnesium homoeostasis may help to sustain a number of enzymatic reactions under saline conditions (Shaul 2002).

### Organic osmolytes

All test grasses accumulated glycinebetaine with increase in salinity, a constitutive trait of plant species included in the Poaceae and Amaranthaceae (Chenopodiaceae) families (Albert 1975; McCue and Hanson 1990; Khan *et al*. 1998; Flowers and Colmer 2008). Proline content increased only in *A. lagopoides* (Sobhanian *et al*. 2010) and was constitutively higher in *S. tremulus* among test grasses. Proline and glycine betaine could be involved in cellular osmotic adjustment, i.e. a reduction of osmotic potential (*ψ*_s_) in plant tissue as a result of vacuolar solute accumulation (Hassine *et al*. 2008). However the relative contribution of these and other osmolytes, such as low molecular carbohydrates and polyols, in cellular osmotic adjustment is debatable. Additional roles include protection and stabilization of thylakoid membranes (Jagendorf and Takabe 2001), PS-II complexes (Murata *et al*. 1992), enzyme structure and activity (Bohnert and Jensen 1996; Mäkelä *et al*. 2000) particularly at high tissue salt concentrations (Wyn Jones 1981). Decreased photosynthetic efficiency (lower WUE) and increased TSS under salt stress could indicate disturbed translocation of sugars and energy starvation in sink tissues of *P. geminatum*. Relative changes in leaf succulence appear to minimize species-specific variations in proline and glycinebetaine concentrations measured in this study; however, the contribution of other osmolytes (such as sugar and polyols not analysed here) in conferring salt resistance cannot be ruled out.

### C/N ratios and NUE

Wide variations in nitrogen content (N) also accounted for proportional variations in NUE of our test grasses (Duarte 1990). Nitrogen deprivation repressed most of the regulatory genes involved in photosynthesis, chlorophyll production and plastid proteins synthesis (Scheible *et al*. 2004). Salt-excreting grasses displayed comparatively lower NUE and C/N ratios (higher N), which correlated well with higher proline and glycine betaine contents compared with non-excreting grasses. High NUE in *P. paspalodes* could reflect its preferential distribution in more stable, naturally anaerobic, saline environments with a low but sustained N supply. However, salt-excreting species appear to prefer well-drained, sandy soils with higher nutrient availability and turnover (Ghannoum 2009; Ghannoum et al. 2011).

## Conclusions

Our hypothesis that there will be distinct variations between salt-excreting versus non-excreting grasses could not be completely proven. Differential responses between salt-excreting and non-excreting grasses occurred only in traits related to succulence, glycinebetaine content and root-to-shoot ion partitioning. This study highlights the relative importance of various combinations of traits which could ultimately help to improve the salt resistance of test grasses and closely associated conventional crops. Similar studies on a number of grasses could provide conclusive evidence for distinct patterns of salt resistance among grasses. Further investigations into drought and flooding stresses would elaborate differences in their responses to abiotic stresses.

## Sources of Funding

This work was supported by the Higher Education Commission of Pakistan.

## Contributions by the Authors

All authors contributed to the writing of the manuscript.

## Conflicts of Interest Statement

None declared.

## Supporting Information

The following Supporting Information is available in the online version of this article –

**Figure S1.** Carbon and nitrogen content of test grasses.

Additional Information
