# Supplementary material for: Excreting and non-excreting grasses exhibit different salt resistance strategies
Source: AoB Plants. 2014 Jul 4;6:plu038. doi: 10.1093/aobpla/plu038 (PMC4224665; doi:10.1093/aobpla/plu038)
Supplement: Additional Information [file supp_6_plu038_index.html]

Excreting and non-excreting grasses exhibit different salt resistance strategies — Additional Information 

# Excreting and non-excreting grasses exhibit different salt resistance strategies

## Additional Information

Additional Information

**Files in this Data Supplement:**

- Additional Information - docx file
