# Supplementary material for: Excreting and non-excreting grasses exhibit different salt resistance strategies
Source: AoB Plants. 2014 Jul 4;6:plu038. doi: 10.1093/aobpla/plu038 (PMC4224665; doi:10.1093/aobpla/plu038)

SUPPLEMENTARY DATA

Supplementary data are available online at [www.aob.oxford-journals.org](http://www.aob.oxford-journals.org) and consist of the following Figure S1: Carbon and nitrogen content of all test grasses.

ACKNOWLEDGEMENTS

This work was supported by the Higher Education Commission of Pakistan. We are thankful to Mr. Tabassum Hussain and Mr. Mohammad Yousuf Adnan for ion analyses in plant samples.


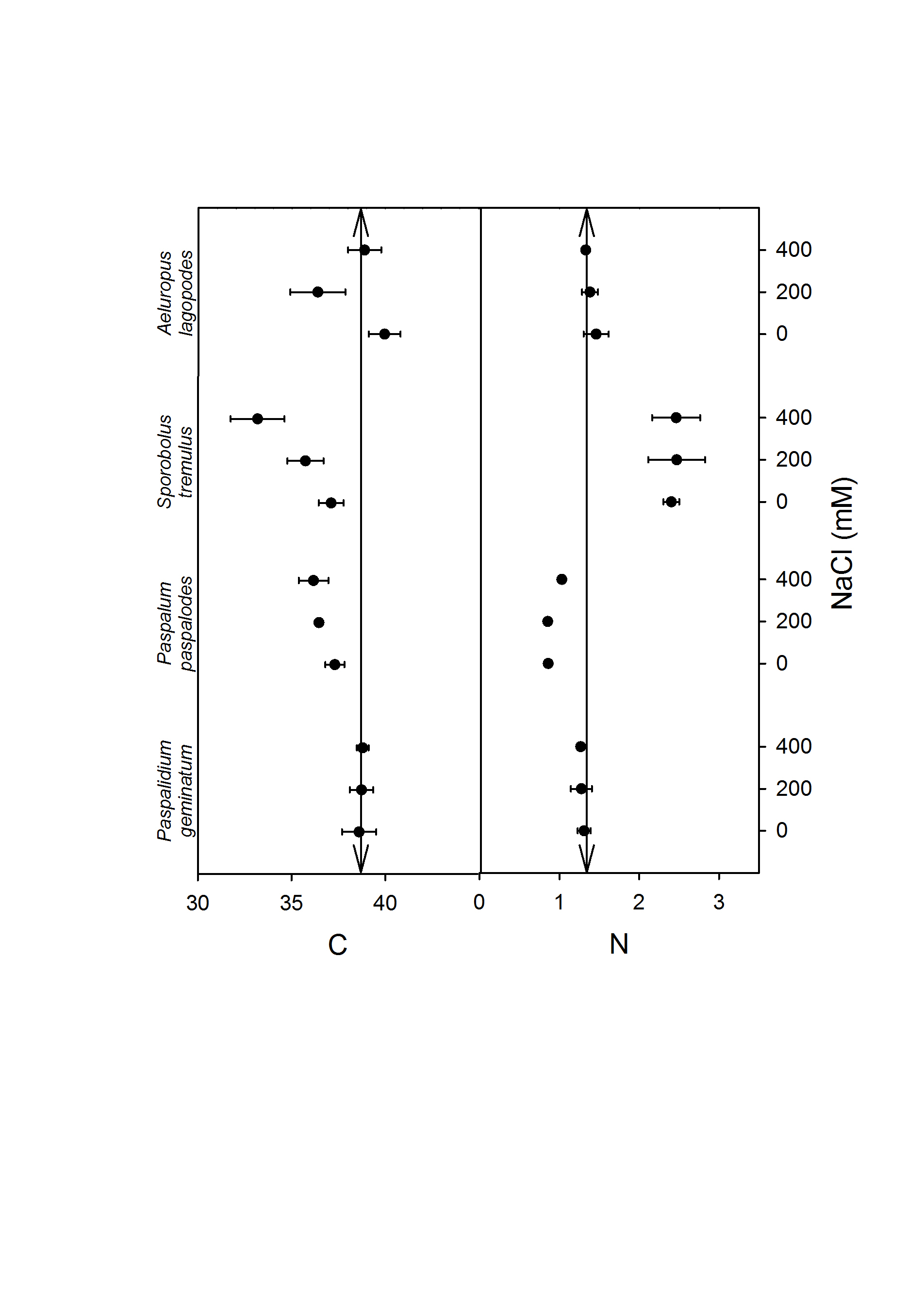

Supplement: Additional Information [file supp_plu038_plu038supp.docx]
